# Supplementary material for: Perivascular Macrophages Convert Physical Wound Signals Into Rapid Vascular Responses
Source: bioRxiv. 2024 Dec 12:2024.12.09.627538. Preprint. [Version 1] doi: 10.1101/2024.12.09.627538 (PMC11661168; doi:10.1101/2024.12.09.627538)
Supplement: Supplement 1 [file NIHPP2024.12.09.627538v1-supplement-1.pdf]

bioRxiv preprint doi: https://doi.org/10.1101/2024.12.09.627538; this version posted December 12, 2024. The copyright holder for this preprint (which was not certified by peer review) is the author/funder, who has granted bioRxiv a license to display the preprint in perpetuity. It is made available under aCC-BY 4.0 International license.

Extended Data Fig. 1

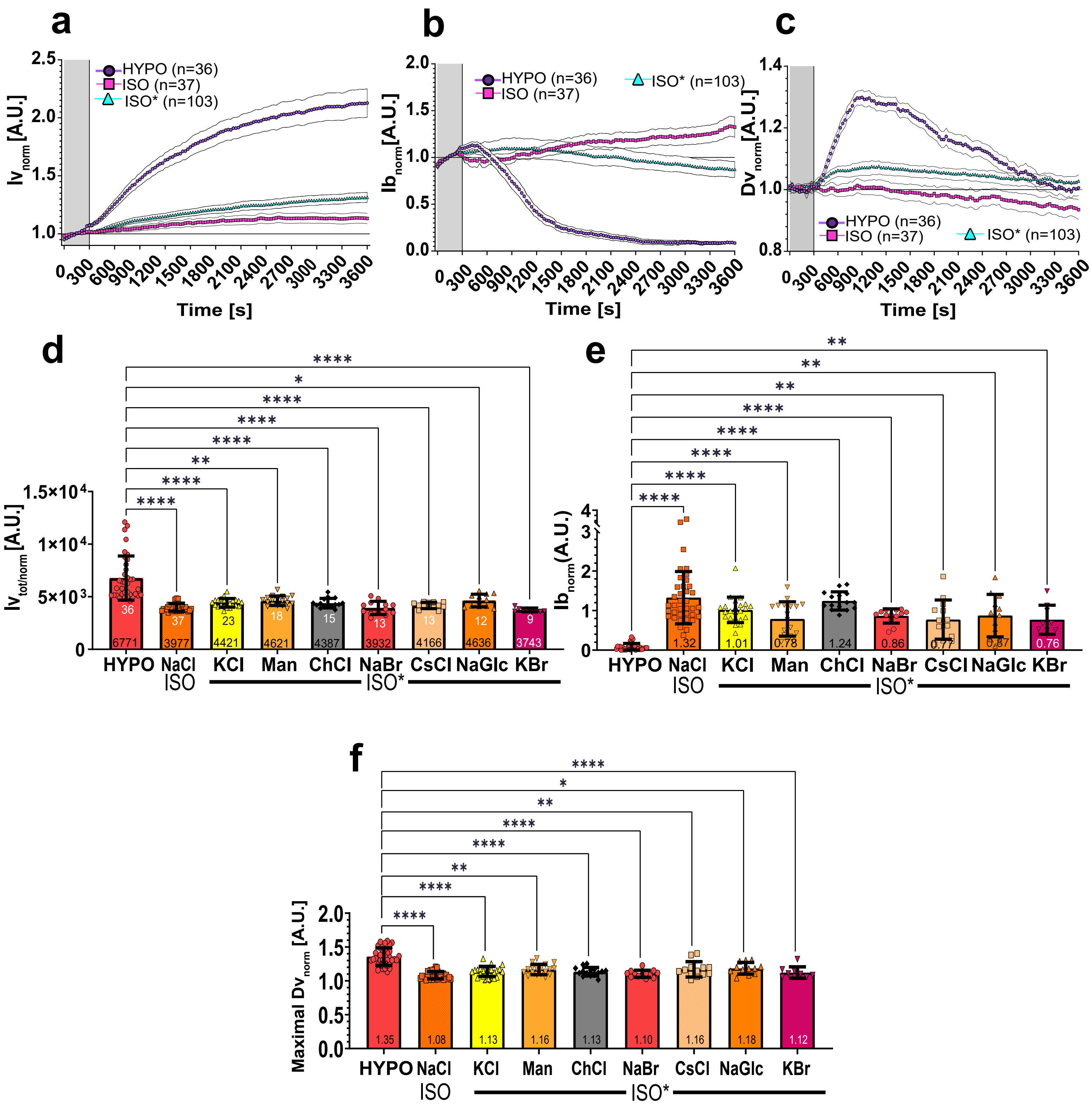

bioRxiv preprint doi: https://doi.org/10.1101/2024.12.09.627538; this version posted December 12, 2024. The copyright holder for this preprint (which was not certified by peer review) is the author/funder, who has granted bioRxiv a license to display the preprint in perpetuity. It is made available under aCC-BY 4.0 International license.

Figure S1. Extended data supporting figure 1. Dynamics of normalized (**a**) vessel leakage ($Iv_{tot/norm}$(T)), (**b**) wound leakage (Ib_{norm} (T)), and (**c**) vessel dilation (Av_{norm}(T)) at the indicated conditions. HYPO, regular E3. ISO, 280 mOsm NaCl in E3. ISO*, pooled isotonic treatments: KCl, potassium chloride. Man, mannitol. ChCl, choline chloride. CsCl, caesium chloride. NaGlc, sodium gluconate. KBr, potassium bromide. NaBr, sodium bromide. The data was normalized to the mean of the first ten frames (grey shaded bar, T= 0-270 s). Error margins, 95% confidence interval of the indicated numbers of animals. Plots of normalized, (**d**) integrated vessel leakage (T=0-3600 s), (**e**) steady state wound leakage (T=3600 s), and (**f**) maximal vessel dilation at the indicated osmolyte conditions. White numbers, animals. Bottom of bar graph numbers, mean of dataset. P values were determined using ANOVA with Dunn's post-hoc. All figure source data and numerical P values are listed in the Supplementary Excel File 1. *P ≤ 0.05, **P ≤ 0.01, ***P ≤ 0.001, ****P ≤ 0.0001. ns, not significant (P > 0.05).

bioRxiv preprint doi: https://doi.org/10.1101/2024.12.09.627538; this version posted December 12, 2024. The copyright holder for this preprint (which was not certified by peer review) is the author/funder, who has granted bioRxiv a license to display the preprint in perpetuity. It is made available under aCC-BY 4.0 International license.

Extended Data Fig. 2

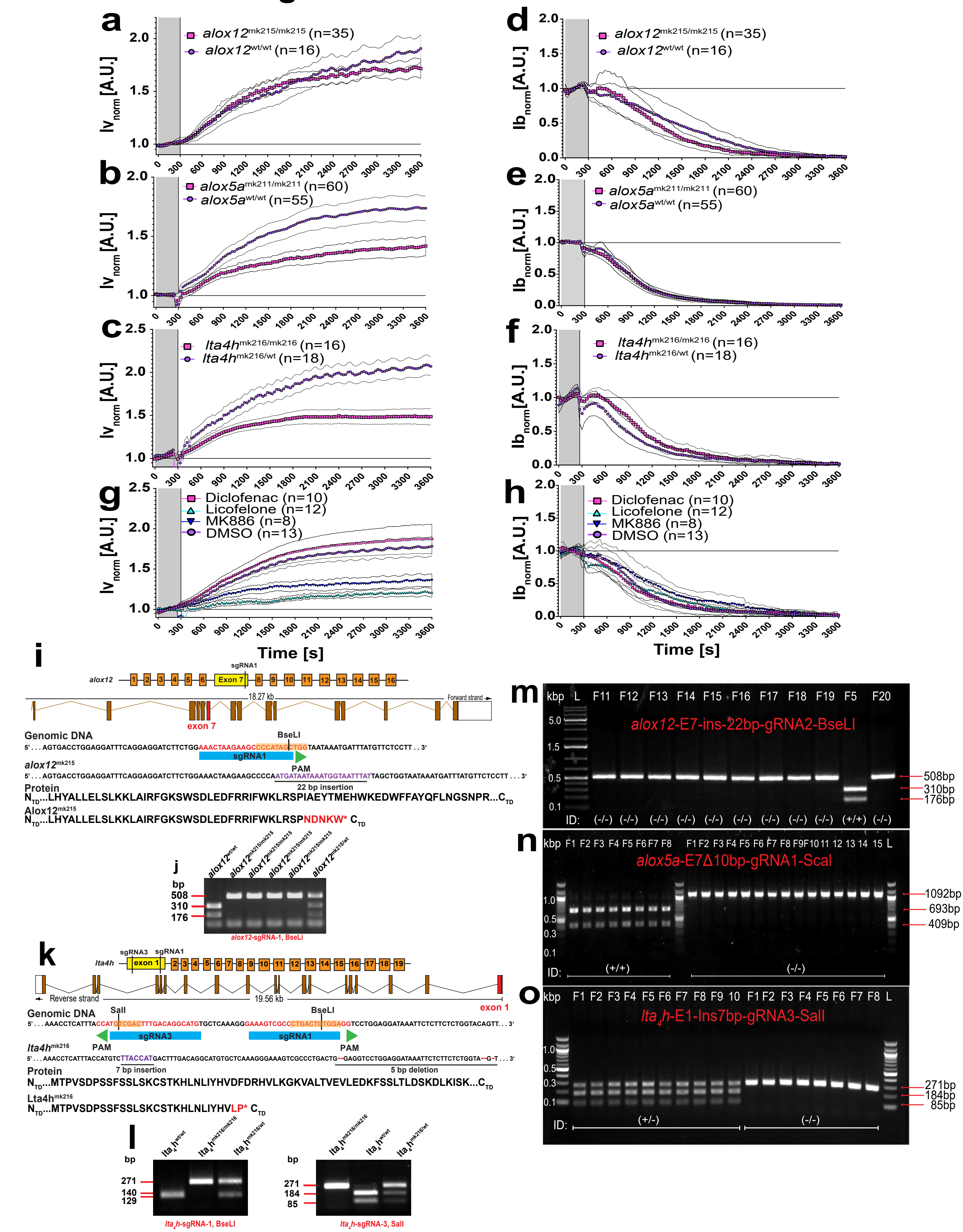

bioRxiv preprint doi: https://doi.org/10.1101/2024.12.09.627538; this version posted December 12, 2024. The copyright holder for this preprint (which was not certified by peer review) is the author/funder, who has granted bioRxiv a license to display the preprint in perpetuity. It is made available under aCC-BY 4.0 International license.

Figure S2. Extended data supporting Fig. 2. Normalized (**a, b, c, g**) vessel- (Iv_{norm}(T)) and (**d, e, f, h**) wound- (Ib_{norm} (T)) leakage dynamics after $ISO_{(NaCl)}$ → HYPO shifting at the indicated genetic and pharmacologic conditions. (**i**) Scheme illustrating the design guide RNAs targeting exon 7 of alox12. The architecture of the exons (brown and red) and introns (black lines) in the alox12 genomic region. The successful gene disruption is validated by BseLI (CCCATAG^CTGG) exon 7 restriction sites (yellow box) on genomic DNA, which is altered in *alox12*$^{mk215/mk215}$ mutants. (**j**) 486 bp polymerase chain reaction (PCR) product of the wildtype allele is cleaved into two fragments (310, 176bp) by BseLI. The mk215 allele carries a genomic 22bp insertion, preventing the targeted BseLI restriction enzyme digest in *alox12*$^{mk215/wt}$ (bp= 508, 310, 176) and *alox12*$^{mk215/mk215}$ (bp=508). (**k**) Scheme illustrating the design of two single guide RNAs targeting exon1 of *lta4h*. The architecture of exons (brown and red) and introns (black lines) in the *lta_4h* genomic region is shown. Successful gene disruption is validated by BseLI (CTGACTG^TGGA) and SalI (G^TCGAC) exon1 restriction sites (yellow box) on genomic DNA, which is altered in *lta_4h*$^{mk216/mk216}$ mutants. (**l**)The 269 bp polymerase chain reaction (PCR) product of the wildtype allele is cleaved into two BseLI (140 and 129 bp) or SalI (184 and 85 bp) fragments. The mk216 allele carries a genomic 7bp insertion and 5bp deletion, preventing the targeted SalI or BseLI restriction enzyme digest in *lta_4h*$^{mk216/wt}$ (bp=271, 140 129; 271, 184 85) and *lta_4h*$^{mk216/mk216}$(271bp). The F0 heterozygotes were identified by the presence of three gel bands corresponding to PCR product enzyme digest of adult tailfins. (**m-o**) Mutations were validated for dextran injected larva after imaging. Representative ethidium bromide DNA with set PCR product restriction enzyme digest with BseLI (bp=310, 176) for *alox12*$^{mk215/mk215}$ (bp=486) and *alox12*$^{wt/wt}$ (bp=310, 176) (**m**), ScaI (bp=693, 409) *alox5a*$^{mk211/mk211}$ (bp=1102) and *alox5a*$^{wt/wt}$ (bp=693, 409) (**n**) and SalI (bp=184, 85bp) *lta_4h*$^{mk216/mk216}$ (bp=271) and *lta_4h*$^{mk216/wt}$ (bp= 271, 184, 85) (**o**), where each well corresponds to a single imaged and analysed larva.

bioRxiv preprint doi: https://doi.org/10.1101/2024.12.09.627538; this version posted December 12, 2024. The copyright holder for this preprint (which was not certified by peer review) is the author/funder, who has granted bioRxiv a license to display the preprint in perpetuity. It is made available under aCC-BY 4.0 International license.

Extended Data Fig. 3

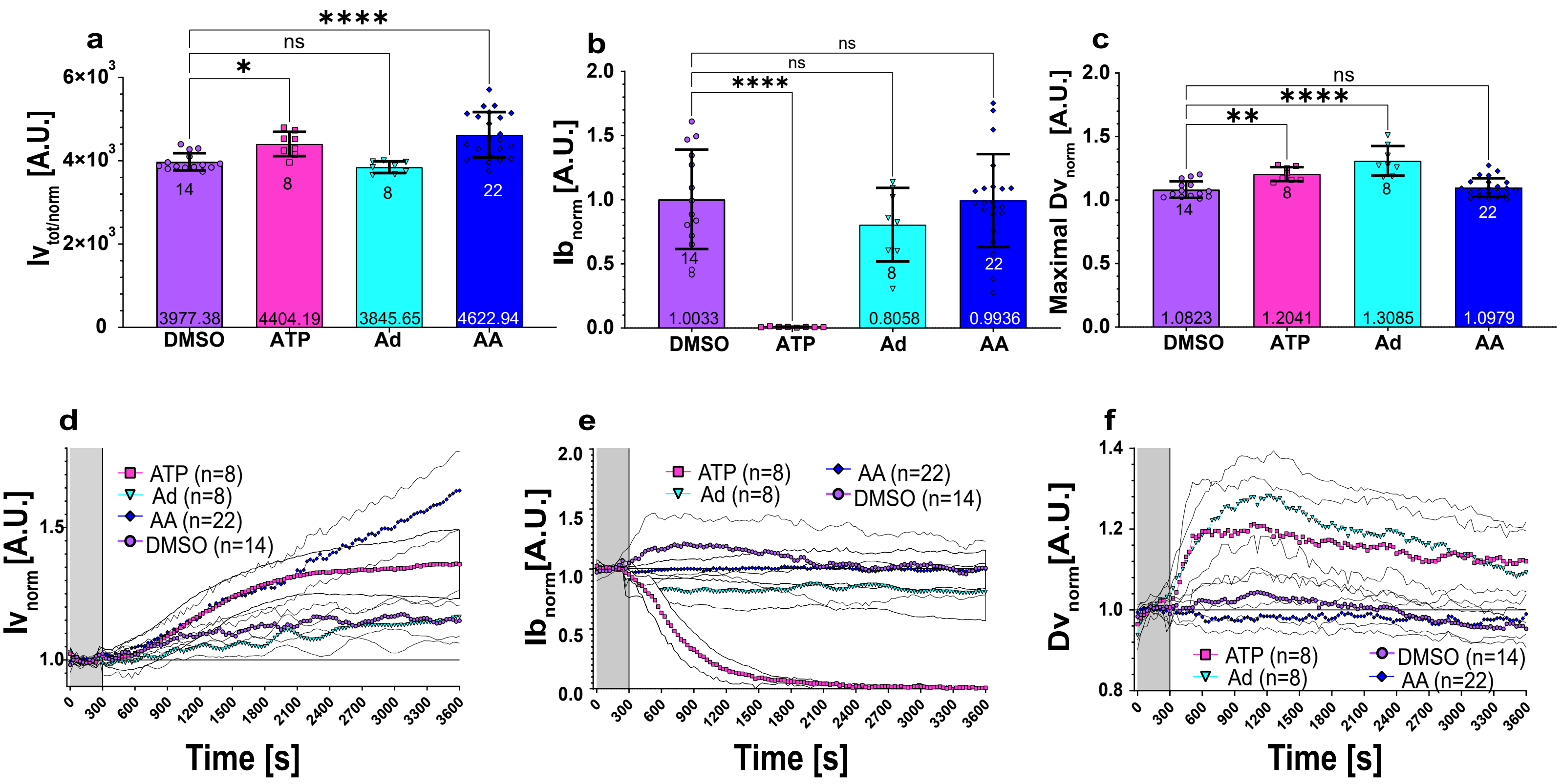

bioRxiv preprint doi: https://doi.org/10.1101/2024.12.09.627538; this version posted December 12, 2024. The copyright holder for this preprint (which was not certified by peer review) is the author/funder, who has granted bioRxiv a license to display the preprint in perpetuity. It is made available under aCC-BY 4.0 International license.

Figure S3. Extended data supporting Fig. 2. Plots of normalized, (**a**) integrated vessel leakage (T=0-3600 s), (**b**) steady state wound leakage (T=3600 s), and (**c**) maximal vessel dilation upon treatment of wounded zebrafish larvae bathed in $ISO_{(NaCl)}$ with the indicated agonists. DMSO, vehicle. ATP, adenosine triphosphate (5 mM). Ad, adenosine (5 mM). AA, arachidonic acid (5 μM). numbers below the error bars, animals. numbers at the bottom of bar graph, mean of dataset. Error bars, SD. Normalized (**d**) vessel leakage ($Iv_{norm}(T)$), (**d, e, f, h**) wound leakage ($Ib_{norm}(T)$), and vessel dilation dynamics after $ISO_{(NaCl)}$ → HYPO shifting with the indicated agonists. Error margin, 95% confidence interval. Note, (a-c) and (d-f) refer to the same data set. P values, one-way ANOVA with Dunnett's post-hoc significance tests. All figure source data and numerical P values are listed in the Supplementary Excel File 1. *P ≤ 0.05, **P ≤ 0.01, ***P ≤ 0.001, ****P ≤ 0.0001. ns, not significant (P > 0.05).

bioRxiv preprint doi: https://doi.org/10.1101/2024.12.09.627538; this version posted December 12, 2024. The copyright holder for this preprint (which was not certified by peer review) is the author/funder, who has granted bioRxiv a license to display the preprint in perpetuity. It is made available under aCC-BY 4.0 International license.

Extended Data Fig. 4

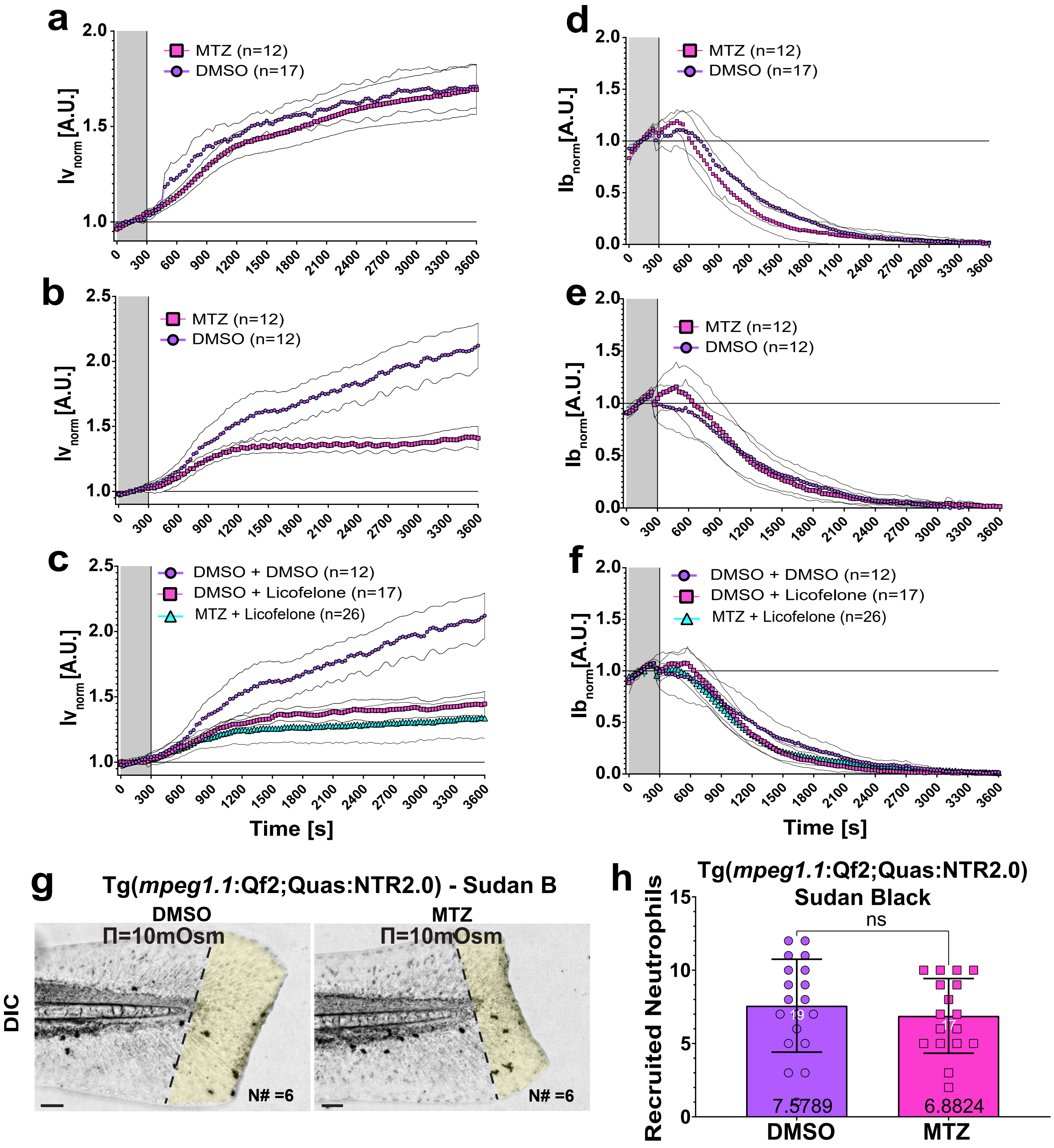

bioRxiv preprint doi: https://doi.org/10.1101/2024.12.09.627538; this version posted December 12, 2024. The copyright holder for this preprint (which was not certified by peer review) is the author/funder, who has granted bioRxiv a license to display the preprint in perpetuity. It is made available under aCC-BY 4.0 International license.

Figure S4. Extended data supporting Fig. 3. Normalized (**a-c**) vessel- ($Iv_{norm}(T)$) and (**d-f**) wound- ($Ib_{norm}(T)$) leakage dynamics after $ISO_{(NaCl)}$ → HYPO shifting at the indicated pharmacologic conditions. Error margin, 95% confidence interval. (**g**) Representative transmitted light image of fixed, control (DMSO) and macrophage depleted (MTZ) tail fins of *mpeg1.1*:Qf2;Quas:NTR2.0 larvae at 90 min post injury. Neutrophils are stained with Sudan Black. N#, number of recruited neutrophils in the tailfin region from notochord to the wound edge (highlighted in yellow). Scale bars, 50 μm. (**h**) Quantification of Sudan Black staining. White numbers, animals. Black numbers, mean of dataset. P values, non-paired two-tailed Student's t-test. The figure source data and numerical P values are listed in the Supplementary Excel File 1. Ns, not significant ($P > 0.05$). Error bars, SD.

bioRxiv preprint doi: https://doi.org/10.1101/2024.12.09.627538; this version posted December 12, 2024. The copyright holder for this preprint (which was not certified by peer review) is the author/funder, who has granted bioRxiv a license to display the preprint in perpetuity. It is made available under aCC-BY 4.0 International license.

Extended Data Figure 5

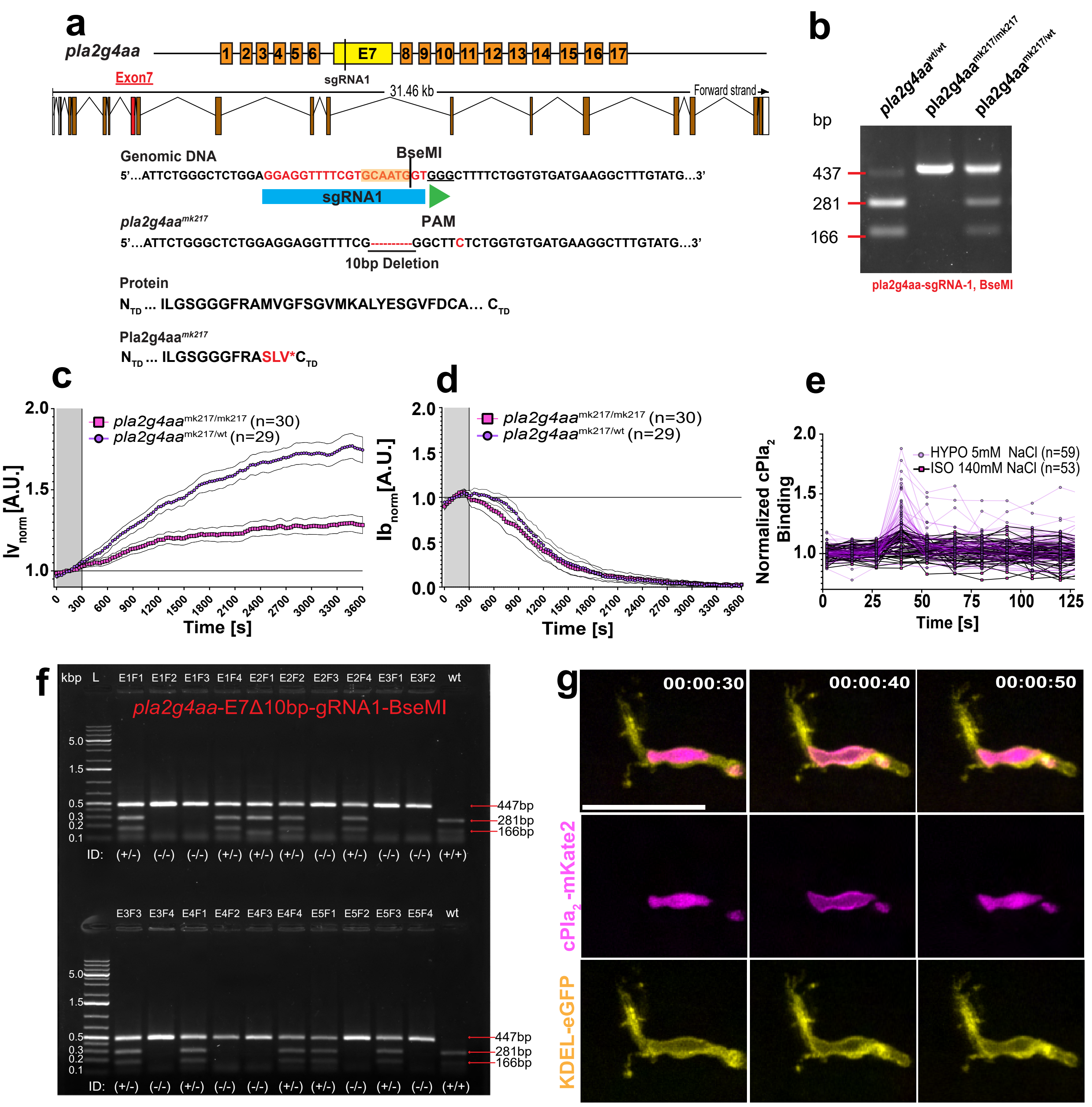

bioRxiv preprint doi: https://doi.org/10.1101/2024.12.09.627538; this version posted December 12, 2024. The copyright holder for this preprint (which was not certified by peer review) is the author/funder, who has granted bioRxiv a license to display the preprint in perpetuity. It is made available under aCC-BY 4.0 International license.

Figure S5. Extended data supporting Fig. 4. (**a**) Scheme illustrating the design of a single guide RNA targeting exon7 of *pla2g4aa* mutant zebrafish. bottom the architecture of exons (brown and red) and introns (black lines) in the zebrafish *pla2g4aa* genomic sequence. Successful gene disruption is validated with BseMI (GCAATG^GT) exon7 restriction palindrome sites (yellow box) on genomic DNA, which is altered in *pla2g4aa*$^{mk217/mk217}$ mutants. (**b**) Representative gel genotyping of CRISPR/Cas9 induced *pla2g4aa* mutations by BseMI restriction digest. The 447 bp (PCR) product of wildtype allele is cleaved by BseMI into two smaller (281 and 166 bp) fragments. The mk217 allele carries 10bp deletion, preventing the targeted BseMI restriction enzyme digest in *pla2g4aa*$^{mk217/mk217}$(437bp) and *pla2g4aa*$^{mk217/wt}$ (437, 281 and 166bp). (**c**) Normalized vessel- (Iv_{norm}(T)) and (**d**) wound- (Ib_{norm} (T)) leakage dynamics after $ISO_{(NaCl)}$ → HYPO shifting at the indicated genetic conditions. Error margin, 95% confidence interval. (**e**) Individual plots underlying Figure 4f. (**f**) Representative genotyping for *pla2g4aa*$^{mk217/wt}$ and *pla2g4aa*$^{mk217/mk217}$ embryos after image acquisition. Red arrows, restriction enzyme cleavage patterns. (**g**) Representative, pseudo-coloured $cPla_2$-mKate2 and endoplasmic reticulum (KDEL-eGFP) fluorescence in a perivascular macrophage before (00:00:30) and after (00:00:40 & 00:00:50) laser injury in hypotonic E3 solution. Timestamp, hh:mm:ss. Scale bars, 25 µm.

Extended Data Figure 6

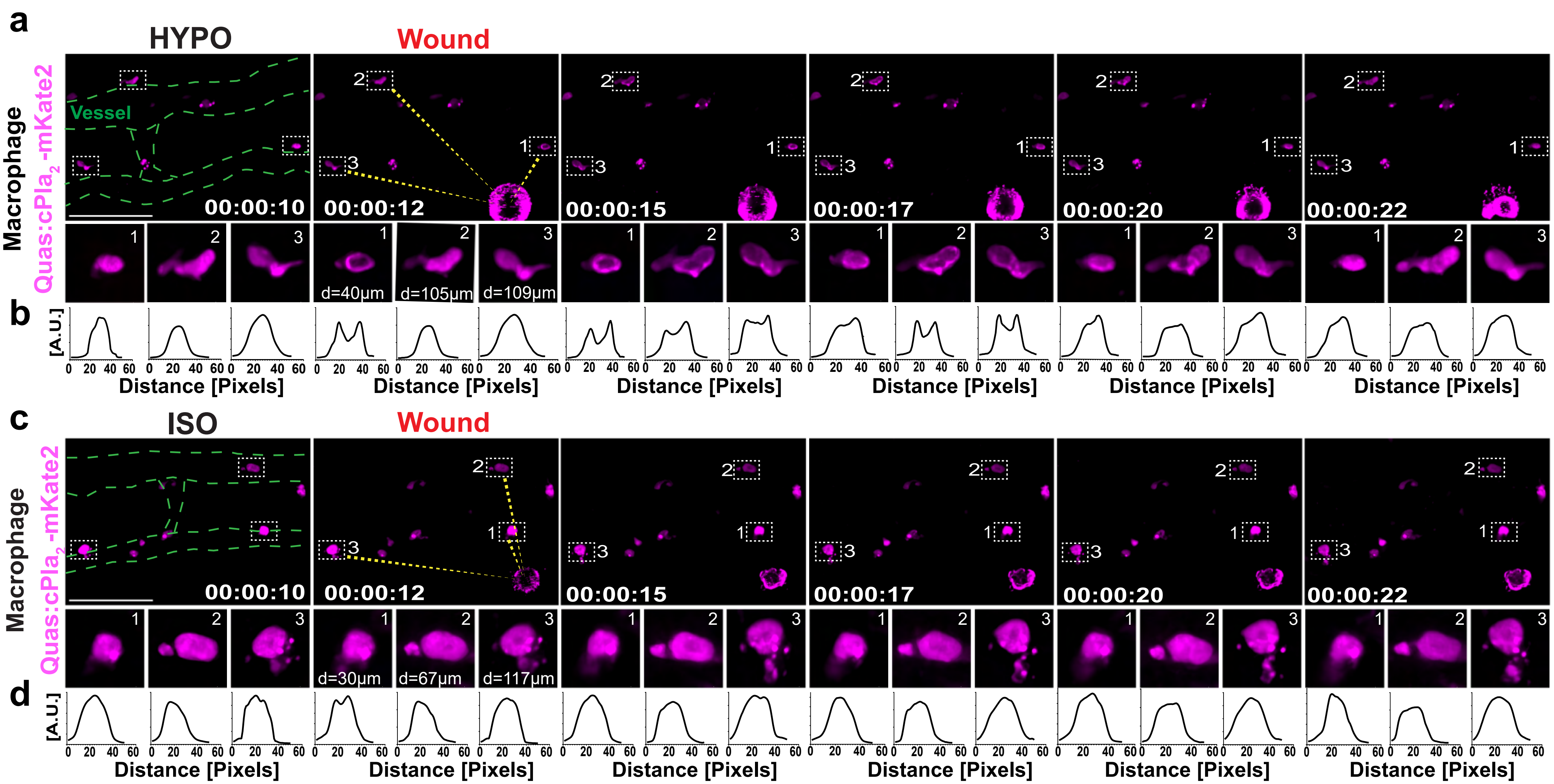

bioRxiv preprint doi: https://doi.org/10.1101/2024.12.09.627538; this version posted December 12, 2024. The copyright holder for this preprint (which was not certified by peer review) is the author/funder, who has granted bioRxiv a license to display the preprint in perpetuity. It is made available under aCC-BY 4.0 International license.

Figure S6. Extended data supporting Fig. 4. Representative two-photon montage of live Tg(*mpeg1.1*:Qf2;Quas:cPla2-mKate2) larvae imaged with rapid temporal resonant scanning (T-step=2.5 s, 6 fps) upon laser injury at T=12 s in (**a**) HYPO and (**c**) $ISO_{(NaCl)}$ E3 bathing solution. Magenta, pseudo-coloured for $cPla_2$-mKate2. Images are representative of three independent wounding experiments. Dashed green line, vessel outline. Timestamp, hh:mm:ss. Scale bars, 50 µm. (**b, d**) Line profiles for $cPla_2$-mKate2 emission([A.U.]) over distance(Pixels) through the macrophages in the numbered ROIs.

bioRxiv preprint doi: https://doi.org/10.1101/2024.12.09.627538; this version posted December 12, 2024. The copyright holder for this preprint (which was not certified by peer review) is the author/funder, who has granted bioRxiv a license to display the preprint in perpetuity. It is made available under aCC-BY 4.0 International license.

Supplementary Videos Legends

Supplementary Video 1. Representative time lapse movie of a 2 dpf zebrafish Tg(*kdrl*:eGFP)-larva showing vessel dilation and vessel permeability upon shifting of bathing solutions from ISO to HYPO. Overexposure of dextran emission is to highlight the bleed out from the wound. Magenta, 70 kDa dextran. Green, vessels. Timestamp, hh:mm:ss. Scale bars, 50 µm.

Supplementary Video 2. Representative time lapse movie of a dextran-injected and -wounded 2 dpf Tg(*kdrl*:eGFP)-larva showing inhibition of vessel dilation and permeability upon shifting of bathing solutions from ISO to ISO_{NaCl}. Magenta, 70 kDa dextran. Green, vessels. Timestamp, hh:mm:ss. Scale bars, 50 µm.

Supplementary Video 3. Representative time lapse movie of a dextran-injected and -wounded 2 dpf Tg(*kdrl*:eGFP)-larva showing mild inhibition of vessel dilation and permeability upon shifting of the bathing solution from ISO to ISO_{ChCl}. Magenta, 70 kDa dextran. Green, vessels. Timestamp, hh:mm:ss. Scale bars, 50 µm.

Supplementary Video 4. Representative time lapse movie of a dextran-injected and -wounded 3 dpf Tg(*lyz*:NTR2.0)-larva showing that neutrophil depletion does not impact vessel- or wound-permeability under hypotonic conditions. Green, neutrophils. Magenta, 70 kDa dextran. Timestamp, hh:mm:ss. Scale bars, 50 µm.

Supplementary Video 5. Representative time lapse movie of a dextran-injected and -wounded 3 dpf Tg(*mpeg1.1*:NTR2.0)-larva showing that absence of macrophages inhibits vessel leakage without impacting wound permeability. Green, macrophage. Magenta dextran 70 kDa. Timestamp, hh:mm:ss. Scale bars, 50 µm.

Supplementary Video 6. Representative time lapse movie of $cPla_2$-mKate2 emission in macrophage nuclei under HYPO and ISO treatments, showing membrane binding after UV-laser wounding (at T= 00:00:40) and rapid recovery (at T= 00:00:50) in HYPO bathing condition. Timestamp, hh:mm:ss. Scale bars, 50 µm.

Supplementary Video 7. Representative two-photon imaging of macrophage $cPla_2$-mKate2 emission with fast temporal acquisition in HYPO and ISO treated zebrafish larvae showing that rapid and reversible $cPla_2$-INM adsorption propagates in wave-like fashion from the site of laser injury (at T=00:00:12). Timestamp, hh:mm:ss. Scale bars, 50 µm.
